# Bactericidal activity and recovery effect of hydroxyl radicals generated by ultraviolet irradiation and silver ion application on an infected titanium surface

**DOI:** 10.1038/s41598-020-65411-4

**Published:** 2020-05-22

**Authors:** Taichi Tenkumo, Kirika Ishiyama, Oleg Prymak, Keisuke Nakamura, Midori Shirato, Toru Ogawa, Makiko Miyashita, Masatoshi Takahashi, Matthias Epple, Taro Kanno, Keiichi Sasaki

**Affiliations:** 1 Division of Advanced Prosthetic Dentistry, Tohoku University Graduate school of Dentistry, 4-1 Seiryo-machi, Aoba-ku, Sendai 980-8575 Japan; 2 Laboratory for Redox Regulation, Tohoku University Graduate School of Dentistry, 4-1 Seiryo-machi, Aoba-ku, Sendai 980-8575 Japan; 3 Inorganic Chemistry and Center for Nanointegration Duisburg-Essen (CeNIDE), University of Duisburg-Essen, Universitaetsstr. 5-7, 45117 Essen, Germany; 4 Division of Dental Biomaterials, Tohoku University Graduate school of Dentistry, 4-1 Seiryo-machi, Aoba-ku, Sendai 980-8575 Japan

**Keywords:** Periodontics, Infection control in dentistry, Medicinal chemistry

## Abstract

This study investigated the bactericidal effect, the underlying mechanisms of treatment, and recovery of biocompatibility of the infected titanium surface using a combination treatment of silver ion application and ultraviolet-A (UV-A) light irradiation. *Streptococcus mutans* and *Aggregatibacter actinomycetemcomitans* were used in suspension and as a biofilm on a titanium surface to test for the bactericidal effect. The bactericidal effect of the combination treatment was significantly higher than that of silver ion application or UV-A light irradiation alone. The bactericidal effect of the combination treatment was attributable to hydroxyl radicals, which generated from the bacterial cell wall and whose yield increased with the silver concentration. To assess the biocompatibility, proliferation and calcification of MC3T3E1 cells were evaluated on the treated titanium surface. The treated titanium screws were implanted into rat tibias and the removal torques were measured 28 days post-surgery. The titanium surface that underwent the combination treatment exhibited recovery of biocompatibility by allowing cellular proliferation or calcification at levels observed in the non-infected titanium surfaces. The removal torque 28 days after surgery was also comparable to the control values. This approach is a novel treatment option for peri-implantitis.

## Introduction

Dental implant treatment is a frequently used prosthodontic practice due to its high success rate^1,2^. However, the incidence of peri-implantitis has increased concurrently^3–5^ with bacterial infection being one of the main causes of peri-implantitis^3,6^. The microstructural topography of an implant’s surface is engineered to facilitate osseointegration. However, infection of the implant surface changes its chemical, mechanical, and topographic characteristics, which leads to a reduction in the implant surface’s biocompatibility^7^. Several treatment methods such as manual treatment using mechanical curettage with ultrasonic devices^8–10^, air powder^11–13^, drug therapy^12–14^, local antiseptics^15^, laser therapy^7,13,16–19^, and photodynamic therapy^7,19–21^ with surgical or non-surgical procedures have been used to facilitate effective debridement or re-osseointegration. However, a gold standard treatment for peri-implantitis has yet to be established.

Silver ions are known to possess antimicrobial activity and commercial products based on this property are used worldwide^22^. In dental treatment, silver diammine fluoride has been used for protection against caries^23^ and several reports have described the mechanisms underlying the antimicrobial activity of silver. Yamanaka *et al*. reported that the silver ion interferes with bacterial metabolism^24^, while Park *et al*. reported that silver ions generate hydroxyl radicals that eliminate bacteria^25^. Recently, Binkley *et al*. reported that electrospun polycaprolactone (PCL) fibres, doped with silver nanoparticles (NPs), reduce bacterial proliferation while supporting osteoblast attachment and proliferation^26^. Slate *et al*. reported that silver application on the dental implant’s surface reduces the risk of infection on biomaterial surfaces^27^. Therefore, the application of silver ions to an infected dental implant surface is expected to demonstrate effective bactericidal activity.

Ultraviolet irradiation has also been used for its bactericidal properties^28^. Ultraviolet irradiation affects bacteria DNA and induces photochemical reactions resulting in bacterial death and/or inhibition of proliferation^29^. The ultraviolet photo-functionalisation procedure has recently been demonstrated to improve commercial titanium surfaces for re-osseointegration^30,31^. Ultraviolet light treatment improved the biocompatibility of titanium surfaces and enhanced osseointegration, both *in vitro* and *in vivo*^32–34^. We have also previously demonstrated that photodynamic therapy, using ultraviolet-A (UV-A) light irradiation with hydrogen peroxide or caffeic acid, has a high bactericidal effect and improves the titanium surface^35–37^.

Based on these findings, a combination treatment consisting of silver ion application and ultraviolet irradiation may serve as an effective peri-implantitis treatment allowing disinfection and improvement of the infected titanium implant surfaces.

In this study, we investigated the bactericidal effect and the underlying mechanism of using a combination treatment of silver ion application and UV-A light irradiation on oral bacterial species, *Aggregatibacter actinomycetemcomitans* and *Streptococcus mutans*. A. actinomycetemcomitans, is one of the bacteria present in a high ratio in peri-implantitis^38^ whereas S. mutans is found primarily in dental plaques^39^ and is a key contributor to the formation of biofilms^39,40^. We also investigated the recovery of titanium surface’s biocompatibility after application of the combination treatment, both *in vitro* and *in vivo*.

## Results

### Bactericidal effect on different bacteria

**Figure 1:**
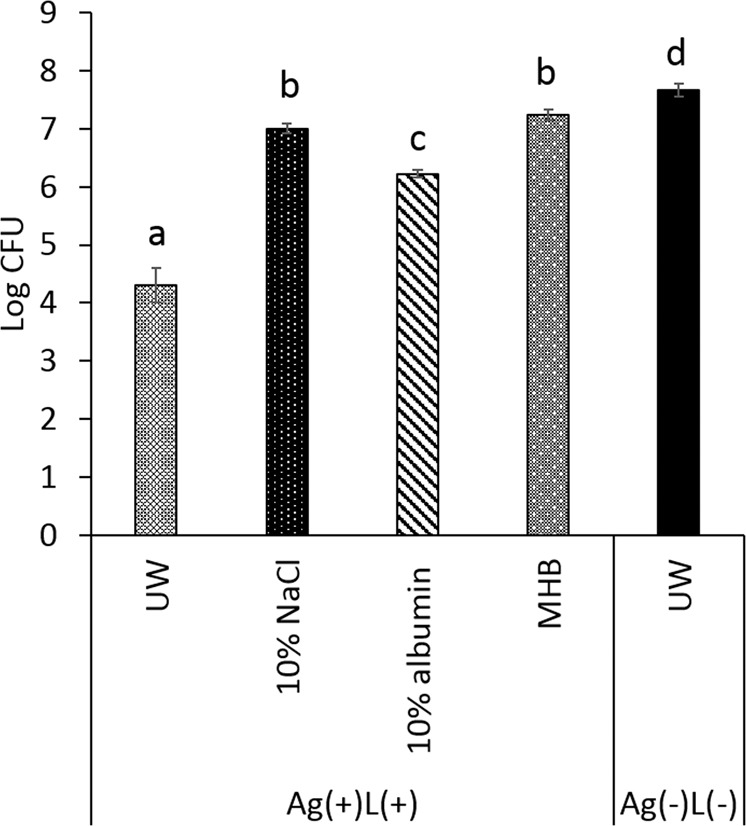
Bactericidal tests using different diluent solutions. The reaction solution obtained after UV-A irradiation was diluted with ultrapure water (UW), 10% NaCl, 10% albumin solution, or MHB solution. Significant differences (p < 0.05) between the groups are denoted by different superscript letters (i.e., bars with the same letter are not significantly different). Ag(+)L(+): Mixture of silver nitrate solution (40 μM)and bacterial suspension (10^7^ CFU/mL) with UV-A irradiation for 1 min, Ag(−)L(−):Mixture of ultrapure water and bacterial suspension (10^7^ CFU/mL) without UV-A irradiation.

The different treatment groups were classified as follows: Ag(+)L(+): Mixture of silver nitrate solution and bacterial suspension followed by UV-A light irradiation, Ag(+)L(−): Mixture of silver nitrate solution and bacterial suspension followed by incubation in a light-shielding box, Ag(−)L(+): Mixture of ultrapure water and bacterial suspension followed by UV-A light irradiation, and Ag(−)L(−): Mixture of ultrapure water and bacterial suspension followed by incubation in a light-shielding box. The protocol for the bactericidal assessment was determined first since the remnant silver ions could affect the results of the bacterial effect test. After the mixture of silver nitrate (40 μM) and the bacterial suspension (10^7^ CFU/mL) was irradiated by UV-A light for 1 min, the reaction solution was diluted with either ultrapure water, 10% NaCl, 10% albumin solution, or 10-fold Mueller Hinton Broth (MHB) solution. The results are shown in Fig. 1. The bactericidal effect in samples diluted with MHB was 7.2 ± 0.1 log CFU, while those in samples diluted with ultrapure water, or 10% albumin solution were 4.3 ± 0.3, and 6.2 ± 0.1 log CFU, respectively, and significantly lower than the value for MHB. This result shows that dilution with ultrapure water, or 10% albumin solution in the bactericidal test did not stop the effects of the remnant silver ions compared to the dilution with MHB. Therefore, the MHB solution was used for diluting the reaction solution in the subsequent bactericidal tests.

**Figure 2:**
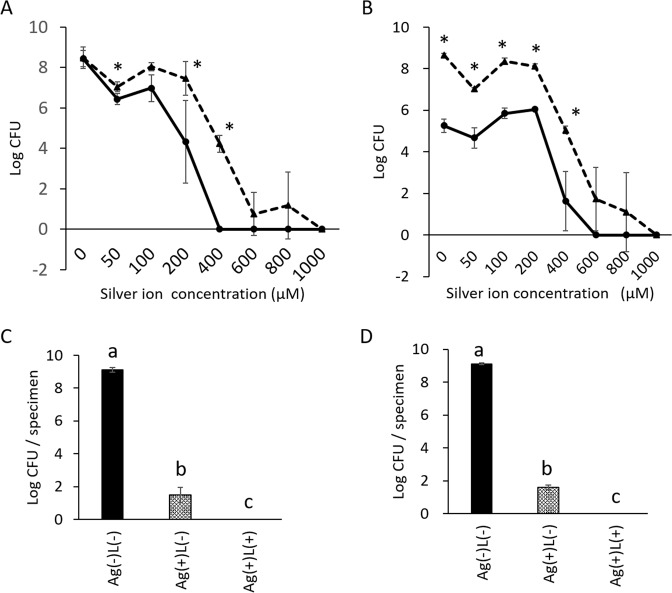
Bactericidal activity of the combination treatment with the silver application and UV-A light irradiation against *S. mutans* or *A. actinomycetemcomitans*. The number of bacteria was determined after each treatment by standard plate counting. (**A**) Colony-forming units (CFUs) for different concentrations of silver nitrate solution in the Ag(+)L(+) and Ag(+)L(−) groups at 1 min for the *S. mutans* suspension and (**B**) for the *A. actinomycetemcomitans* suspension (⚫) Ag(+)L(+), (▴) Ag(+)L(−). Values and error bars indicate the mean and standard deviation, respectively. Statistically significant differences are shown, p < 0.05 (*). (**C**) CFU with 1  mM silver nitrate solution in the Ag(+)L(+) and Ag(+)L(−) groups (irradiance: 1000 mW/cm^2^, treatment time: 1 min) for the *S. mutans* biofilm and (**D**) the *A. actinomycetemcomitans* biofilm. Significant differences (p < 0.05) between the groups are denoted by different superscript letters (i.e., bars with the same letter are not significantly different).

The bactericidal effect of each treatment (silver nitrate (0-1000 μM) and the bacterial suspension (10^7^ CFU/mL)) is shown in Fig. 2. Both, Ag(+)L(+) and Ag(+)L(−) treatments exhibited a bactericidal effect depending on the silver nitrate concentration. For *S. mutans*, 400 µM silver nitrate solution completely eradicated the bacteria in Ag(+)L(+), while the corresponding concentration in Ag(+)L(−) was 1000 µM. The bactericidal effect of Ag(+)L(+) was significantly higher than that of Ag(+)L(−) for silver nitrate concentrations over 50 µM. For *A. actinomycetemcomitans*, both treatments exhibited bactericidal effects. The silver concentrations inducing bacterial eradication in Ag(+)L(+) and Ag(+)L(−) were 600 µM and 1000 µM, respectively. The bactericidal effect of Ag(+)L(+) was significantly higher than that of Ag(+)L(−). For *A. actinomycetemcomitans*, the effect of Ag(−)L(+) was significantly higher than that of Ag(−)L(−), while no significant difference was observed for *S. mutans*.

**Figure 3:**
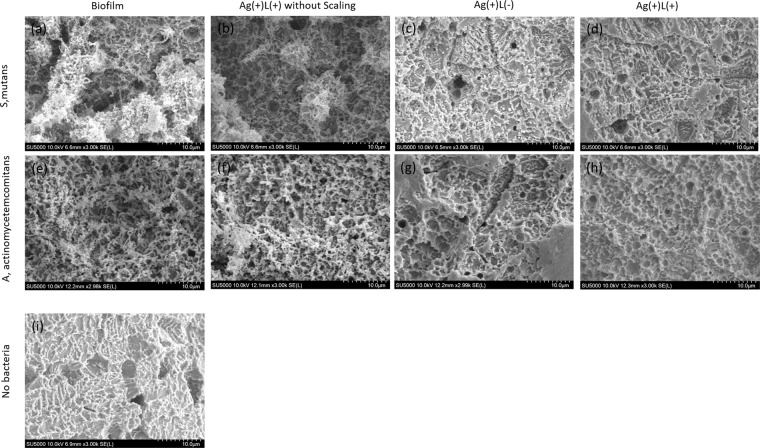
SEM images of the condition of the titanium surface. (**a**) Infected by *S. mutans* for 48 h. (**b**) Infected by *S. mutans* followed by Ag(+)L(+) treatment without ultrasound scaling. (**c**) Infected by *S. mutans* followed by Ag(+)L(−) treatment after ultrasound scaling. (**d**) Infected by *S. mutans* followed by Ag(+)L(+) treatment after ultrasound scaling. (**e**) Infected by *A. actinomycetemcomitans* for 48 h. (**f**) Infected by *A. actinomycetemcomitans* followed by Ag(+)L(−) treatment without ultrasound scaling. (**g**) Infected by *A. actinomycetemcomitans* followed by Ag(+)L(−) treatment after ultrasound scaling. (**h**) infected by *A. actinomycetemcomitans* followed by Ag(+)L(+) treatment after ultrasound scaling. (**i**) Sandblasted and acid-etched with no infection by bacteria.

The SEM images show that the sandblasting and acid-etching treatments resulted in a rough titanium surface (Fig. 3i). Biofilms of both bacterial species formed successfully on the titanium surfaces (Fig. 3a,e). The biofilms of both bacterial species remained on the titanium surface after application of silver nitrate solution and UV-A irradiation (Ag(+)L(+)), without any ultrasound scaling (Fig. 3b,f). In the Ag(+)L(−) group, the biofilm was not observed on the titanium surface, however, particle-like amorphous deposits were observed (Fig. 3c,g). The biofilm was not observed in the Ag(+)L(+) group (Fig. 3d,h).

Based on the SEM results, the bactericidal test for biofilm was performed in the Ag(+)L(−) and Ag(+)L(+) groups after the ultrasound scaling. For the bactericidal effect on the biofilm, no CFUs were observed with a silver concentration of 1 mM in the Ag(+)L(+) group for *S. mutans* and *A. actinomycetemcomitans*, while the corresponding values for Ag(+)L(−) were 1.5 ± 0.5 and 1.6 ± 0.1 log CFU, respectively. This indicates a significant difference between the Ag(+)L(−) and Ag(+)L(+) treatments.

### Analysis of hydroxyl radical generation

**Figure 4:**
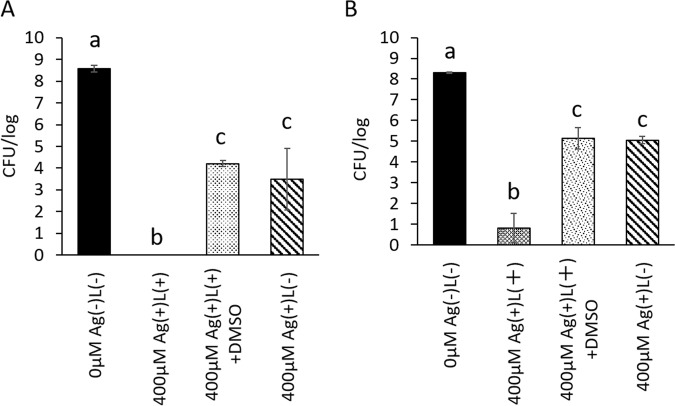
Results of bactericidal assessments in the Ag(+)L(+) group with or without DMSO (a reactive oxygen species scavenger agent) in (**A**) *S. mutans*, (**B**) *A. actinomycetemcomitans*. Ag(+)L(+) + DMSO: Mixture of bacterial suspension and silver nitrate solution (400 μM) was irradiated with UV-A light for 1 min with 1.4 M DMSO. Ag(+)(L + ): without DMSO, and Ag(+)L(−): without UV-A light irradiation and DMSO. Ag(−)L(−): initial count. Values and error bars indicate the mean and standard deviation, respectively. Significant differences (p < 0.05) between the groups are denoted by different superscript letters (i.e., bars with the same letter are not significantly different).

To investigate whether the bactericidal effect of the combination treatment was caused by hydroxyl radicals, bactericidal tests were performed with or without DMSO as a reactive oxygen species scavenger. Figure 4 shows the results of the bactericidal tests with and without DMSO at a silver ion concentration of 400 μM. The colony-forming unit value in group Ag(+)L(+) without DMSO was 0 ± 0 log for *S. mutans* and 0.8 ± 0.7 log for *A. actinomycetemcomitans*, while the corresponding values with DMSO were 4.2 ± 0.1 and 5.1 ± 0.5 log, respectively. A significant difference was observed between the values obtained with and without DMSO in the Ag(+)L(+) group. The corresponding values in the Ag(+)L(−) group without DMSO were 3.5 ± 1.4 log for *S. mutans* and 5.0 ± 0.2 log for *A. actinomycetemcomitans*, and the values with DMSO were 4.2 ± 0.1 log and 5.1 ± 0.5 log, respectively. These values were closer to those of Ag(+)L(+) with DMSO.

**Figure 5:**
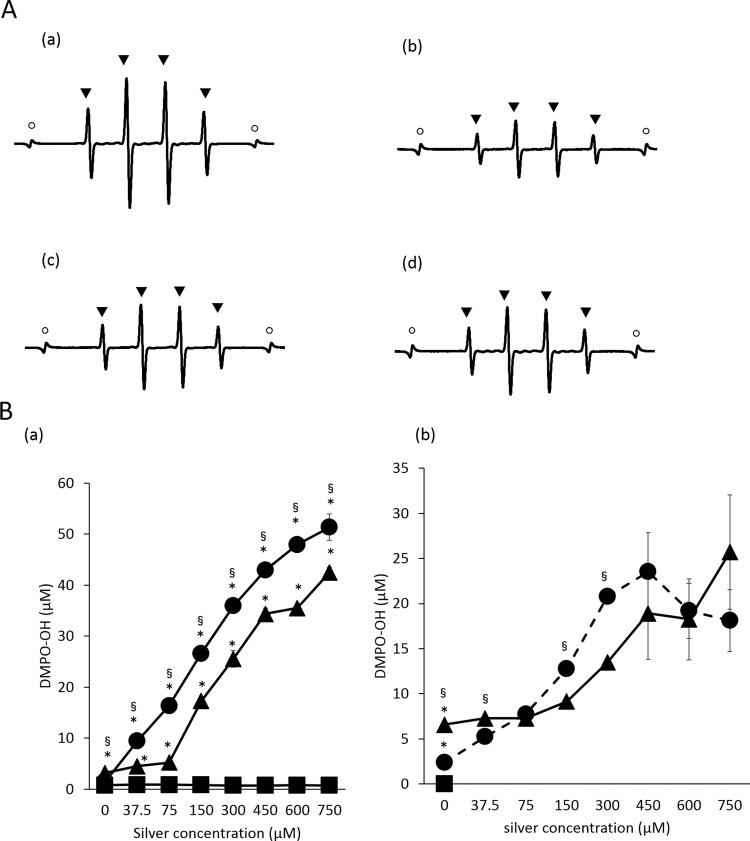
(**A**) Analysis of hydroxyl radicals generated by UV-A irradiation of silver ion solution (2 mM) with (a): dispersion of *S. mutans*, (b): dispersion of *A. actinomycetemcomitans*, (c): biofilm of *S. mutans*, and (d): biofilm of *A. actinomycetemcomitans*. Hydroxyl radicals were qualitatively and quantitatively analysed using an electron spin resonance (ESR)-spin-trapping technique with DMPO as the spin trap agent. ○ and ▴ show the MnO marker and DMPO-OH, respectively. (**B)** Hydroxyl radicals generated by silver nitrate solution application and UV-A light irradiation for bacteria. Hydroxyl radicals were qualitatively and quantitatively analysed using the ESR-spin-trapping technique with DMPO. (**a**) The yield of DMPO-OH generated by silver nitrate solution application with UV-A light irradiation to *S. mutans* suspension (•)*, A. actinomycetemcomitans* suspension (▴), ultrapure water(×), and without bacteria (◾). (**b**) The corresponding values for *S. mutans* biofilm (•), *A. actinomycetemcomitans* biofilm (▴), and ultrapure water without biofilm (◾). Values and error bars indicate the mean and standard deviation, respectively. Statistically significant differences are shown, p < 0.05 (*).

Fig. 5A shows the representative ESR spectra of 5,5-dimethyl-1-pyrroline N-oxide (DMPO)-OH and the spin adduct of the hydroxyl radical generated by 2 mM silver nitrate solution and UV-A (365 nm) irradiation with dispersions or biofilms of different bacteria. The presence of DMPO-OH was confirmed by hyperfine coupling constants (hfcc) of aN = aH = 1.49 mT22. The patterns of ESR spectra in all groups were consistent with that of DMPO-OH. The ESR patterns in all groups were similar to that of hydroxyl radicals.

Figure 5B shows the yield of DMPO-OH generated by different concentrations of silver nitrate application with or without UV-A light irradiation to bacterial suspensions or biofilms. The generated DMPO-OH in the Ag(+)L(+) group with the bacterial dispersion ranged from 11 to 48 μM for *S. mutans* and from 4 to 42 μM for *A. actinomycetemcomitans*, while the corresponding values without bacteria were approximately 0.3-0.9 µM. The values in the Ag(−)L(−) group for *S. mutans* and *A. actinomycetemcomitans* were 1.0 and 0.7 μM, respectively. For the biofilms of *S. mutans* and *A. actinomycetemcomitans*, the generated DMPO-OH levels in the Ag(+)L(+) group ranged from 5.3 to 23.5 μM and from 7.3 to 25.7 μM, respectively. The corresponding values for the Ag(−)L(+) group were 2.4 and 6.6 µM, respectively and those for the Ag(−)L(−) with *S. mutans* and *A. actinomycetemcomitans* biofilms were 0.03 and 0.02 μM, respectively.

To further investigate the part of bacteria that reacted with silver nitrate solution and UV-A light irradiation, the hydroxyl radical levels generated from each bacterial cellular fraction were measured (Table 1). Hydroxyl radicals were generated from all cellular fractions but the yields of the generated hydroxyl radical from each bacterial cellular fraction were different. The hydroxyl radical levels generated from the cell wall were 2.3 ± 1.0 μM in *S. mutans* and 5.3 ± 0.7 μM in *A. actinomycetemcomitans*, which were higher than those in the other cellular fractions.

**Table 1:** Hydroxyl radical levels generated by the silver application and UV-A light irradiation with each bacterial cellular fraction.

DMPO-OH (μM)	Cell wall	Cell membrane	Cytoplasm	
*S. mutans*	2.3 ± 1.0	0.3 ± 0.4	0.2 ± 0.1	
*A. actinomycetemcomitans*	5.3 ± 0.7	0.1 ± 0.0	0.4 ± 0.3	
LPS (1 μg / mL)				87.7 ± 5.1
Peptidoglycan (1 μg / mL)				2.0 ± 0.7

**Figure 6:**
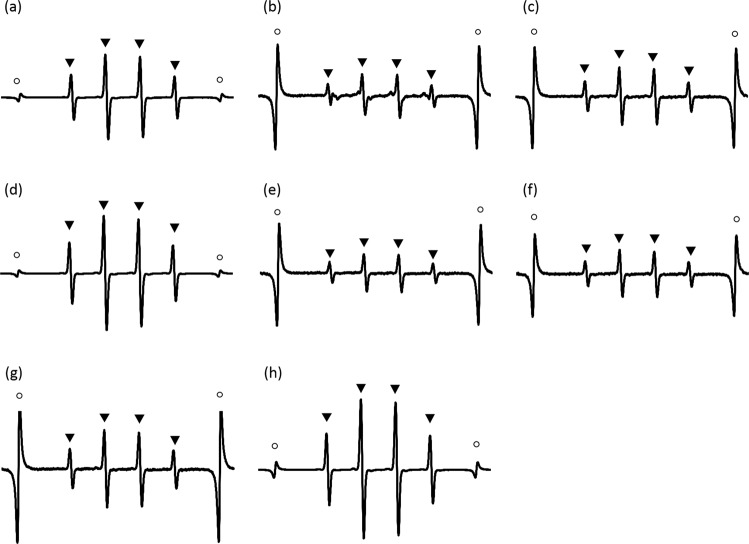
The representative ESR spectra for DMPO-OH generated by silver nitrate solution and UV-A irradiation with the dispersion of *S. mutans* containing the (**a**) cell wall, (**b**) cell membrane, and (**c**) cytoplasmic fraction, and the dispersion of *A. actinomycetemcomitans* containing the (**d**), cell wall, (**e**) cell membrane, and (**f**) cytoplasmic fraction. Spectra for (**g**) peptidoglycan (1 μg/mL) and (**h**) LPS (1 μg/mL). Since the ESR spectra generated from the cell membrane, cytoplasmic fraction and peptidoglycan were smaller than those generated from the cell wall, they are presented at higher magnification. Note that the difference in the MnO marker intensity. ○ and ▴ shows the MnO marker and DMPO-OH, respectively. The presence of DMPO-OH was confirmed by hyperfine coupling constants (hfcc) of aN = aH = 1.49 mT22.

Figure 6 shows the representative ESR spectra pattern from each cellular fraction. The ESR patterns for hydroxyl radicals in the cell wall, cell membrane, and cytoplasm were identical. Furthermore, the hydroxyl radical levels generated by LPS (1 μg/mL) and peptidoglycan (1 μg/mL), which are components of the cell wall, were 87.7 ± 5.1 μM and 2.0 ± 0.7 μM, respectively. The ESR patterns for hydroxyl radicals in LPS and peptidoglycan were identical.

### Biocompatibility of the combination treatment *in vitro* and *in vivo*

**Figure 7:**
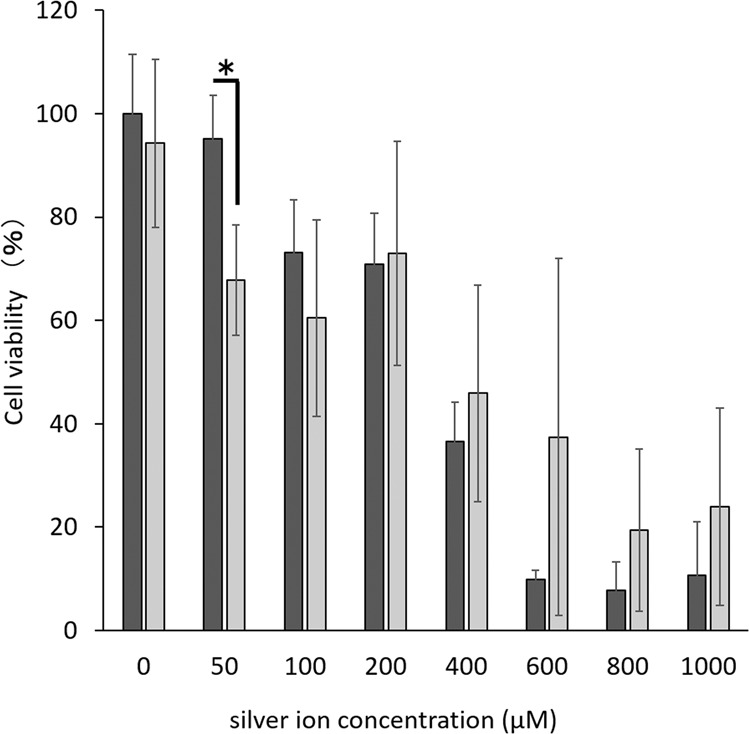
Comparison of the cell viability of the combination treatment (Ag(+)L(+)) or only silver ion application (Ag(+)L(−)) at different silver ion concentrations. Only silver nitrate application () or combination treatment (). Values and error bars indicate the mean and standard deviation, respectively. Statistically significant differences are shown, p < 0.05(*).

Figure 7 shows the cell viability of the combination treatment. The cell viability with silver ion treatment decreased depending on the silver ion concentration. On the other hand, UV-A light irradiation alone reduced the cell viability to 94% ± 16%. The viability in group Ag(+)L(+) was comparable to that in group Ag(+)L(−), except at 50 μM. No significant difference was observed between Ag(+)L(+) and Ag(+)L(−) groups from 100 to 1000 μM.

**Figure 8:**
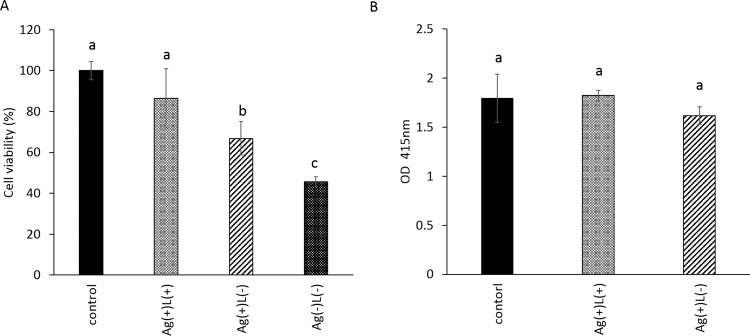
Comparison of the cell compatibility of the titanium surface that underwent the combination treatment (Ag(+)L(+)) or silver ion application (Ag(+)L(−)) after ultrasound scaling on *A. actinomycetemcomitans* biofilm or non-infected titanium surface. (**A**) cell proliferation, (**B**) cell calcification. Significant differences (p < 0.05) between the groups are denoted by different superscript letters (i.e., bars with the same letter are not significantly different).

The *in vitro* findings for cell biocompatibility of the titanium surface after the combination treatment following ultrasound scaling on biofilm of *A*. *actinomycetemcomitans* are shown in Fig. 8. The proliferation of MC3T3E1 cells on the titanium surface treated by Ag(+)L(+) recovered to the levels of the control (Fig. 8A). The corresponding value for Ag(+)L(−) was significantly lower than that of the control and Ag(+)L(+) groups. The value for Ag(−)L(−) was significantly lower than that of the other groups. On the other hand, calcification in the Ag(+)L(+) and Ag(+)L(−) groups at 28 days was similar to that in the control group (Fig. 8B). No significant differences were observed among the groups.

For the *in vivo* investigation of tissue biocompatibility, a biofilm-infected titanium screw was prepared. After the prepared titanium screw received the combination treatment following ultrasound scaling, the treated titanium screw was implanted into rat tibia and the removal torque was measured at 28 days post-surgery. The results of the removal torque test are shown in Table 2. The removal torque value in the Ag(+)L(+) group was comparable to that in the normal group (sandblasted and acid-etched titanium screw without bacterial infection implanted into the tibia).

**Table 2:** Removal torque test.

	Removal torque (cN/mm)
Normal	10.3 ± 1.5
Ag(+)L(+)	10.1 ± 1.0

**Figure 9:**
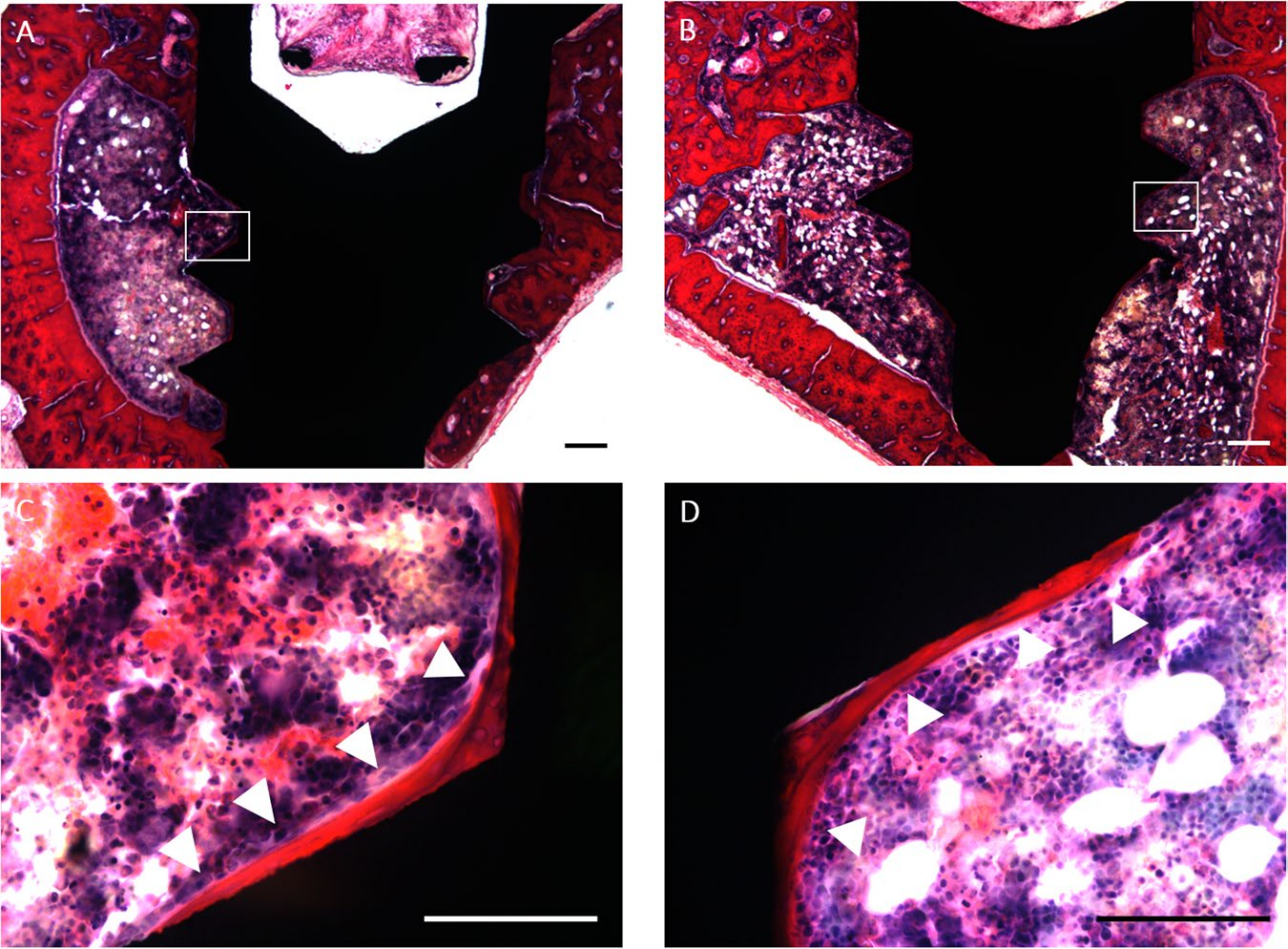
Histologic images of the tissue surrounding the implanted titanium screws 28 days after implantation in the tibia of rats. Histological assessment of the normal titanium screw (**A**,**C**) and the titanium screw in the Ag(+)L(+) group (**B**,**D**) after staining with haematoxylin and eosin (**H**–**E**). Scale bars: 100 µm (**A**,**B**) and 50 µm (**C**,**D**).

Figure 9 shows the non-decalcified tissue around the implant at 28 days after surgery. No severe inflammation, necrosis, or drainage was observed in both groups. Partial osseointegration at the bone-implant interface was observed in both groups.

## Discussion

The main causative organism behind peri-implantitis has not yet been identified. *Aggregatibacter. actinomycetemcomitans, Porphyromonas gingivalis, Treponnema denticola*, and *Tannerella forsythia* have all been detected at high ratios in peri-implantitis^38^. On the other hand, *S. mutans* has been detected in dental plaque^39^. While the biofilm is comprised of several bacterial species, *S. mutans* is a key contributor for the formation of biofilms^39,40^. *S. mutans* synthesises adhesive extracellular matrix, which allows the assembled biofilms to firmly attach onto the tooth and titanium surfaces^41,42^. Furthermore, we have previously demonstrated that *S. mutans* is more resistant to the antimicrobial activity of hydroxyl radicals^43^. The *S. mutans* in this study, therefore, provides more insight into the full potential of the bactericidal effect of this treatment.

*Finally, A. actinomycetemcomitans* is a gram-negative bacterium, whereas *S. mutans* is a gram-positive bacterium. In this study, we opted to study the two different gram stain bacterium to gain further insights into different outcomes of each strain when subjected to a combination treatment of silver ion application and UV-A irradiation.

### Bactericidal effect of the combination of silver application and UV-A light irradiation

The 1 mM (169.8 ppm) silver ion concentration inhibited bacterial proliferation to log CFU 0 and this concentration is substantially higher than those reported previously. This difference could be attributed to the exposure time of silver ion and UV-A irradiation. Since shorter treatment times are better for disinfection treatment, the silver exposure time was set at 1 min in this study. In other reports, the complete inhibitory concentrations of silver ion ranged from 0.08 to 18.9 μM^22,44–47^. However, the exposure times of silver application were longer than that used in this study and no previous report has used a silver ions exposure time smaller than 1 min. For example, Choi *et al*. reported that the complete eradication concentration of silver ion against *Escherichia coli* was approximately 4.2 μM but their exposure time was 24 hours^45^.

The application of silver ions exhibited bactericidal effects in a silver ion concentration dependence manner, which is consistent with the previous reports^22^. UV-A irradiation is also known to possess a bactericidal effect and the resistance to UV-A irradiation differs across bacterial species^28^. A UV-A irradiation for 1 min exhibited bactericidal effects against *A. actinomycetemcomitans* but not *S. mutans* in this study. Nakamura *et al*. reported that UV-A irradiation for 4 min on an *S. mutans* suspension exhibited a 1−log reduction of CFU^36^. This may be due to the short irradiation time. However, the combination treatment with silver ion application and UV-A light irradiation exhibited a higher bactericidal effect than that of silver ion application or UV-A irradiation alone. This result indicates that the addition of UV-A irradiation to silver ion’s application enhanced the bactericidal effect. On the other hand, the biofilm remained on the titanium surface after the combination treatment with no ultrasound scaling, whereas no biofilm remained in SEM observations obtained following ultrasound scaling. In bactericidal activity’s assessment, the combination treatment followed by ultrasound scaling showed a significantly higher bactericidal effect than the effect of silver ion application alone after ultrasound scaling. These results suggest that the use of the combination treatment alone was not enough to remove the biofilm but was useful as an adjunctive treatment for peri-implantitis.

### Bactericidal mechanism of the combination of silver application and UV-A irradiation

Next, we investigated the mechanism underlying the bactericidal effect of this combination treatment. Park *et al*. reported that silver ions generate hydroxyl radicals^25^. Meanwhile, Song *et al*. noted that UV-A irradiation generates reactive oxygen species in water^28^. The generated hydroxyl radical induces oxidative damage in DNA, proteins, and cell membranes and leads to delays in growth^48,49^. Based on these reports, we hypothesised that the enhanced bactericidal effect of the combination treatment might be attributable to active oxygen generation. Therefore, assessments of bactericidal activity with or without the reactive oxygen species scavenger, DMSO, were performed in this study. The bactericidal effect of the combination treatment decreased with DMSO and the ESR investigations showed that generated active oxygen molecules were hydroxyl radicals. Thus, the bactericidal effect of the combination treatment may be attributable to hydroxyl radical generation.

The yield of hydroxyl radicals generated by the combination treatment increased with the silver ion concentration and was approximately 10-40-fold of that obtained with the silver application alone. However, the yield of hydroxyl radicals generated with the combination of a silver application and UV-A irradiation without bacteria was significantly smaller than that generated in the presence of bacteria. This suggests that all the 3 elements of this combination—bacteria, silver ions, and UV-A irradiation—were essential for a high yield of hydroxyl radical generation. Furthermore, the yield of the generated hydroxyl radicals from the bacterial cell wall was higher than that from the cell membrane or cytoplasm in both bacterial species. This suggests that the silver ion attaches to the bacterial cell wall and generates hydroxyl radicals under UV-A light irradiation. Furthermore, the combination treatment generated a high yield of hydroxyl radicals with LPS which suggests that the combination treatment captures free LPS and removes it via hydroxyl radicals, and may be a useful treatment for periodontitis.

### Biocompatibility of the combination treatment with the silver application and UV-A irradiation

ROS are generated by the inflammatory cells such as the macrophage or neutrophil in chronic inflammation and the generated ROS are related to the progress of periodontitis^50^. In general, the generated hydroxyl radicals were promptly disappeared^51^, whereas the ROS generation continued due to inflammatory cells in periodontitis. The results of the cell-cytotoxicity test show that the combination treatment did not enhance the cytotoxicity of silver ions. Miyayama *et al*. reported that exposure of the cells to 2.5 μM silver nitrate for 24 h reduced the cell viability by half^51^. However, the IC_50_ of the combination treatment and silver ion application with 1 min exposure was within the range of 200-400 μM in this study. This difference is attributable to the exposure time. Foldjerg *et al*. reported that the cytotoxicity of silver ions was dependent on the exposure time and concentration^52^. Therefore, the cytotoxicity of ROS generated in this combination treatment is believed to be limited.

Treatments using the mechanical or chemical approach have been used for cleaning infected implant surfaces; however, these methods induce surface alternation and result in a decrease of the implant surface’s biocompatibility^7^. Therefore, the recovery of the titanium surface’s biocompatibility after disinfection is an important factor for peri-implantitis treatment. The proliferation and calcification of MC3T3E1 cells recovered to a level comparable with that of the non-infected titanium surface. This indicates that the biocompatibility of the titanium surface was recovered by the combination treatment and potentially induced bone re-osseointegration. *In vivo*, the removal torque force of the titanium screw that underwent the combination treatment was consistent with that of a non-infected titanium screw. Histological observations showed that the newly formed bone attached directly to the titanium surface in both groups and the osseointegration was observed after silver application and UV-A light irradiation treatment, similar to the non-infected titanium group. These results indicate that silver ion application and UV-A light irradiation allowed recovery of the infected titanium implant surface and induced re-osseointegration. Yamada *et al*. reported that UV light irradiation recovered the hydrophilic titanium surface and improved cell compatibility^33^. We also report that UV-A irradiation with H_2_O_2_ recovered the hydrophilic titanium surface and improved cell compatibility^37^. Thus, UV-A irradiation might have changed the surface condition hydrophilicity, which may have contributed to the recovery of biocompatibility in this study.

In this study, UV-A irradiation intensity was set at 1000 mW/cm^2^, in line with our previous report^53^. In dental treatment, 465 nm LED at an irradiance of up to 4000 mW/cm^2^ has been used to cure the light-curing composite resin in oral cavities^54^. Cell viability of the combination of UV-A at 1000 mW/cm^2^ and silver nitrate is statistically comparable to that without UV-A irradiation in this study. This indicates that UV-A irradiation at 1000 mW/cm^2^ for 1 min is not high enough to affect cell proliferation.

UV-A has been used with an exogenous photosensitiser such as Photochemotherapy for not only skin diseases including atopic dermatitis or psoriasis^55^ but also for oral mucosa manifestations of chronic graft-versus-host disease^56,57^. Therefore, we believe that the use of an adequate dose of UV-A is acceptable if the treatment efficacy prevails the risk of an adverse effect. However, when the combination treatment of UV-A irradiation and silver is applied to peri-implantitis, the bone around the implant is also exposed to UV-A. DNA damage of UV-A irradiation is different when in the chromatin conformation. Moreover, the safety range of the dose of UV-A to the bone and the effect of the combination treatment should be further investigated in a peri-implantitis model.

## Materials and methods

### Bacterial cultures

Two species of oral bacteria, *Streptococcus mutans* JCM5705 (RIKEN BioResource Center, Wako, Japan) and *Aggregatibacter actinomycetemcomitan*s JCM2434 (RIKEN BioResource Center, Wako, Japan) were used in this study. Cultures were grown anaerobically using AneroPack (Mitsubishi Gas Chemical Company, Tokyo, Japan) in a brain heart infusion (BHI) broth (Becton Dickinson Labware, Franklin Lakes, NJ, USA) or BHI broth with 10 g/L of yeast extract (BHI-YE) (Oxoid, Hampshire, UK) at 37 °C for 24 h. The prepared bacterial suspension was diluted in sterile saline and contained approximately 1 × 10^8^ colony-forming units (CFUs)/mL, in line with our previous report^35^. For the biofilm-related test, the bacterial suspension was seeded on the titanium surface or plastic well and incubated in BHI-YE for *Aggregatibacter actinomycetemcomitans* or BHI with 1% sucrose (Kanto chemical, Japan) (BHI-S) for *Streptococcus mutans* at 37 °C for 24 h in anaerobic conditions.

### Titanium surface treatment

Commercially pure titanium disks (diameter, 5 mm: thickness, 2 mm: Grade 4; TB550, Nishimura, Fukui, Japan) were sandblasted and acid-etched, according to our previous report^37^, to obtain a rough surface similar to the commonly used commercial dental implants. Briefly, sandblasting was performed with 250 µm alumina particles at a blasting-pressure of 0.4 MPa. The specimens were acid-etched in 49% sulphuric acid for 1 h at 60 °C and were the cleaned by ultrasound-cleaning in ultrapure water (10 min), acetone (10 min), and finally ultrapure water (10 min). After cleaning, the specimens were autoclaved for 15 min at 121 °C. The samples were prepared 1 day before use and stored in ultrapure water.

### Bactericidal assay

#### Establishment of a protocol for determination of the treatment effect

The prepared bacterial suspension (100 μL; final concentration, 10^7^ CFU/mL) was mixed with 100 μL of silver nitrate solution (40 μM) in a 96-well cell culture plate. After mixing, the sample was irradiated with UV-A light (Omnicure LX400 + ; Lumen dynamics, Japan) at an irradiation intensity of 1000 mW/cm^2^ for 1 min. The reacted solution was diluted 2-fold with ultrapure water, 10% sodium chloride (NaCl) (Wako, Japan), 10% albumin solution (Wako, Japan), or 10-fold Mueller Hinton Broth (MHB) (BBL Brain Heart Infusion, BD, USA) solution to stop the bactericidal effect of the remnant silver ions. The MHB solution was prepared as follows: 370 g of BHI powder was dissolved into 1 L of ultrapure water and autoclaved for 15 min at 121 °C and then kept for 1 h at room temperature. These groups were designated as “Ag(+)L(+) UW,” “Ag(+)L(+) 10% NaCl,” “Ag(+)L(+) 10% albumin,” and “Ag(+)L(+) MHB,” respectively. As a control, the bacterial suspension was mixed with ultrapure water and incubated in a black box for 1 min and diluted 2-fold with ultrapure water and designated as “Ag(−)L(−) UW.” Next, the treated solution was diluted 10–10^5^-fold with saline, after which 10 μL of the diluted solution was seeded on BHI agar (Oxoid, Basingstoke, UK) for determination of the treatment effect. The agar plates were anaerobically cultured at 37 °C for 48 h followed by colony enumeration for the determination of CFU/mL.

#### Bacterial suspension

In this step, 100 μL of the prepared bacterial suspension was mixed with 100 μL of different concentrations of silver nitrate solution (100, 200, 400, 800, 1200, 1600, and 2000 μM) or ultrapure water in a 96-well cell culture plate. The final concentrations of silver nitrate solutions were 50, 100, 200, 400, 600, 800, and 1000 μM (8.5, 17.0, 33.97, 67.9, 101.9, 135.9, and 169.8 ppm, respectively). The final concentration of the bacterial suspension was 10^7^ CFU/mL. After mixing, the sample was irradiated with UV-A light (365 nm) (Omnicure LX400 + ; Lumen Dynamics, Japan) at an irradiation intensity of 1000 mW/cm^2^ or kept in a light-shielding box for 1 min. The samples were classified into 4 groups depending on the treatment method as follows; Ag(+)L(+): mixing silver nitrate solution and bacterial suspension followed by UV-A light irradiation, Ag(+)L(−): mixing silver nitrate solution and bacterial suspension followed by incubation in a light-shielding box, Ag(−)L(+): mixing ultrapure water and bacterial suspension followed by UV-A light irradiation, Ag(−)L(−): mixing ultrapure water and bacterial suspension followed by incubation in a light-shielding box. The reacted solution was diluted 2-fold with 10-fold MHB solution. CFU/mL was finally evaluated by colony enumeration after 48 h as described above. The initial bacterial count (inoculum size) was also evaluated.

To determine the mechanism underlying the bactericidal effect of the combination treatment with silver nitrate and UV-A irradiation, 100 μL of the prepared bacterial suspension was mixed with 100 μL of silver nitrate solution (final concentration; 400 μM [33.97 ppm]) with 1.4 M Dimethyl sulfoxide (DMSO) as a reactive oxygen species scavenger or ultrapure water in a 96-well cell culture plate. After mixing, the sample was irradiated with UV-A light at 1000 mW/cm^2^ irradiation intensity or kept in the light-shielding box for 1 min. Next, the reacted solution was subjected to the same protocol. All tests were performed as 3 independent assays.

#### Biofilm on the titanium surface

The biofilm on the titanium surface was prepared as above described. The culture solution of the prepared biofilm was removed, and the titanium was washed with PBS twice. After removing PBS, the titanium plate was cleaned with an ultrasound scaler plastic tip (Varios V-P10; Nakanishi, Japan) using an ultrasound scaler device (Varios 970; Nakanishi, Japan) for 1 min. The titanium plate was then immersed in 500 μL of 1000 μM (169.8 ppm) silver nitrate solution and irrigated with UV-A light at an irradiance of 1000 mW/cm^2^ or kept in a light-shielding box for 1 min. These groups were designated as “Ag(+)L(+)” or “Ag(−)L(+),” respectively. The treated titanium plate was washed with PBS twice and incubated in 300 µL of a mixture of type I collagenase (4 mg/mL; Thermo Fisher Scientific, Japan) and Disperse (2 mg/mL; Thermo Fisher Scientific, Japan) for 2 h under shaking culture at 37 °C to extract the remaining bacteria from the titanium surface. Subsequently, 250 µL of the reacted solution was seeded onto BHI agar and cultured at 37 °C for 48 h, followed by colony enumeration for determination of the CFU/mL value. As a control group, the infected titanium was immersed into ultrapure water for 1 min and subjected to the same procedure. This group was designated as “Ag(−)L(−).” The extracted solution was diluted 10-fold with sterile saline, and then, 10 μL of the diluted solution was seeded onto BHI agar.

#### Scanning electron microscopic observation

The titanium disks and the biofilm were prepared as described above. The prepared biofilm on the titanium desk was subjected to ultrasound scaling with an ultrasound scaler plastic tip for 1 min, which was followed by immersion into 1 mM silver nitrate solution with either irradiation with UV-A light an irradiance of 1000 mW/cm^2^ or incubation in a light-shielding box for 1 min; these groups were designated as “Ag(+)L(+)” and”Ag(+)L(−),” respectively. As a control group, the prepared biofilm on the titanium desk was immersed into 1 mM silver nitrate solution and irradiated with UV-A light for 1 min and designated as an “Ag(+)L(+) without scaling.” To observe the titanium surface, the treated titanium disk was examined using a scanning electron microscopy (SEM; SU-5000; Hitachi, Japan) at 10 Kv following carbon-palladium alloy sputtering.

### Analysis of hydroxyl radical generation

#### Bacterial suspension

The yield of hydroxyl radicals generated by the combination treatment with silver ion application and UV-A light irradiation was analysed by an electron spin resonance (ESR) spin-trapping technique following a previously published study^58^. Briefly, 75 µL of silver nitrate solutions of different concentrations (100, 200, 400, 800, 1200, 1600, 2000 μM) or ultrapure water were mixed with 50 µL of 5,5-dimethyl-1-pyrroline *N*-oxide (DMPO) (Labotec, Tokyo, Japan) in a well of a 96-well plate, and 75 μL of the bacterial suspension of *S. mutans* or *A. actinomycetemcomitans*, adjusted to approximately 10^8^ CFU/mL, was added. The final concentration of silver nitrate was 0, 37.5, 75, 150, 300, 450, 600, or 700 μM (6.4, 12.7, 25.5, 51.0, 76.4, 102.0, or 127.4 ppm, respectively,) and that of DMPO was 75 mM. The prepared solution was subjected to irradiation with UV-A light at an irradiance of 1000 mW/cm^2^ or incubated in a light-shielding box for 1 min. Each reacted solution was analysed in an X-band ESR spectrometer (JES-FA-100, JEOL, Tokyo, Japan) and the ESR spectrum of each sample was recorded. The concentration of DMPO-OH (hydroxyl radicals trapped by DMPO) was determined using the Digital Data Processing software (JEOL). Tests were performed as 3 independent assays.

#### Biofilm

The *S. mutans* or *A. actinomycetemcomitans* biofilms were prepared in a 96-well plastic cell culture plate as above described. The supernatant of the prepared biofilm was removed and 75 µL of ultrapure water, 75 µL of silver nitrate solutions of different concentrations, and 50 µL of DMPO (final concentration 75 mM) were added. The final concentration of silver nitrate was 0, 37.5, 75, 150, 300, 450, 600, or 700 μM (6.4, 12.7, 25.5, 51.0, 76.4, 102.0, or 127.4 ppm). The mixed solution was irradiated with UV-A light (irradiation intensity: 1000 mW/cm^2^) or kept in a light-shielding box for 1 min and subjected to the same procedure.

#### Bacterial cellular fraction

The bacterial cellular fraction was prepared following the report by Kumode *et al*.^59^. Briefly, bacterial suspensions of *S. mutans* or *A. actinomycetemcomitans* were incubated in BHI broth or BHI-YE at 37 °C for 24 h. The prepared bacterial suspension was centrifuged at 13,000 × *g* for 10 min at 4 °C. The supernatant was removed, and the precipitate was re-suspended in protoplast buffer (50 mM Tris-HCl, 5 mM EDTA, 5 mM NaCl, and 25% sucrose at pH 7.5) followed by addition of lysozyme (final concentration, 1 mg/mL) and incubated at 37 °C for 16 h. The obtained bacterial suspension was centrifuged at 13,000 × *g* for 10 min at 4 °C, and the supernatant was collected as the cell wall component of bacteria. The remaining precipitate was re-suspended in ultrapure water and centrifuged at 13,000 × *g* for 10 min at 4 °C, and the supernatant was collected as the bacterial cell membrane component while the precipitate was collected as the cytoplasmic component. All obtained components were lyophilised and resuspended in ultrapure water. A mixture of 75 μL of each cellular fraction (10 µg/mL), 75 µL of 2 mM (339.6 ppm) silver nitrate solution, and 50 µL of 75 mM DMPO was irradiated with UV-A light (irradiation intensity: 1000 mW/cm^2^) for 1 min. The ESR spectrum of each prepared suspension was analysed.

### Biocompatibility

#### Cellular cytotoxicity of the combination of silver application and UV-A light irradiation

The MC3T3-E1 mouse osteoblastic cell line provided by RIKEN Cell Bank (Tsukuba, Japan) was used. Cells were cultured in a cell culture flask using Dulbecco’s (D) modified Eagle’s medium (α-MEM; Nakalai Tesque, Kyoto, Japan) supplemented with 10% foetal bovine serum (FBS; Thermo Fisher Scientific, Waltham, MA, USA), 100 U/mL penicillin, and 0.1 mg/mL streptomycin (1% P/S; Wako Pure Chemicals Industries, Osaka, Japan) at 37 °C under a 5% CO_2_ atmosphere. The MC3T3E1 cells were trypsinised and seeded on the treated titanium disk in 96-well plates at a density of 5 × 10^3^ cells per well. The plates were incubated overnight at 37 °C under a 5% CO_2_ atmosphere. The culture medium was removed and washed with PBS. Next, 100 μL of each silver nitrate solution with a different concentration (100, 200, 400, 800, 1200, 1600, and 2000 μM) was added into the wells and irradiated with UV-A light for 1 min. The final concentrations of silver nitrate solutions were 50, 100, 200, 400, 600, 800, and 1000 μM. The reaction solution was then removed and 100 μL of fresh D-Medium was added, and the mixture was incubated for 3 h at 37 °C under a 5% CO_2_ atmosphere. Cell viability was determined by the 3-(4,5-dimethylthiazol-2-yl)-2,5-diphenyltetrazoliumbromide (MTT; Sigma, Japan) assay according to our previously reported protocol^60^. The culture medium was replaced with MTT solution (final concentration 1 mg/mL), followed by incubation for 1 h at 37 °C under a 5% CO_2_ atmosphere. The MTT solution was replaced with DMSO, followed by incubation for 30 min at room temperature. The absorbance of the reacted solution at 570 nm was measured using a microplate reader (Spectra MAX 190; Molecular Devices, Japan). As a control, untreated MC3T3E1 cells at a density of 5×10^3^ cells per well were immersed into ultrapure water in a light-shielding box for 1 minute. The ultrapure water was removed and 100 μL of fresh D-Medium was added, and the cells were incubated for 3 h at 37 °C under a 5% CO_2_ atmosphere. The absorption of the cells was normalised to that of the control.

#### Cell proliferation assay and cell calcification assay

The titanium disks and the biofilm were prepared using *A. actinomycetemcomitans* as above described. The infected titanium desk was subjected to ultrasound scaling for 1 min, followed by immersion into 2 mM (339.6 ppm) silver nitrate solution with either irradiation with UV-A light at 1000 mW/cm^2^ or incubation in a light-shielding box for 1 min. The treated titanium samples were washed with PBS and designated as the “Ag(+)L(+)” and “Ag(+)L(−)” groups, respectively. For the negative control group, the titanium desk was cleaned with ultrasound scaling for 1 min, immersed into ultrapure water, incubated in a light-shielding box for 1 min, and named “Ag(−)L(−).” For the positive control group, the non-infected titanium desk treated without application of silver nitrate and UV-A irradiation was prepared and named the control group.

The MC3T3E1 cells were trypsinised and seeded on the treated titanium disk in 48-well plates at a density of 3 × 10^4^ cells per well. The plates were incubated at 37 °C under a 5% CO_2_ atmosphere for 3 days for cell proliferation analysis or 28 days for cell calcification analysis. Cell proliferation was determined by the MTT assay. Cell calcification was determined by Alizarin red staining. One gram of Alizarin red S (Nacalai tesque, Japan) and 0.1 mL of 28% ammonium solution (Nacalai tesque, Japan) were diluted with 100 mL of ultrapure water. Both prepared solutions were mixed, and the pH was adjusted to 6.4. The cells were washed with PBS and fixed with methanol for 20 min. After removing the methanol, the cells were sufficiently washed with PBS, followed by addition of 250 μL of the prepared Alizarin red solution in room temperature for 30 min. After removing the Alizarin red solution, the cells were sufficiently washed with PBS and 250 μL of 5% formic acid at room temperature was added, followed by shaking for 10 min. Subsequently, 100 μL aliquots were taken for spectrophotometric analysis with an absorbance microplate reader at 415 nm.

#### *In vivo* removal torque test

A titanium screw (diameter, 1.6 mm: length, 6.12 mm: Fukuoka Seimitu, Japan) was sandblasted, acid-etched, and autoclaved as described above. After the formation of the biofilm of *A. actinomycetemcomitans* on the titanium screw, the infected titanium screw was subjected to ultrasound scaling for 1 min, which was followed by immersion into 2 mM (339.6 ppm) silver nitrate solution. Subsequently, the mixed solution was irradiated with UV-A light at an irradiance of 1000 mW/cm^2^. In the control group, after the titanium screw was sandblasted, acid-etched and autoclaved, the screw was stored in ultrapure water without any bacterial infection.

Wistar rats (8 weeks old) were used in this experiment in accordance with the guidelines for the care and use of laboratory animals of Tohoku University. The rats were anaesthetised with an intraperitoneal injection of medetomidine (Domitor; 0.375 mg·kg^−1^ bodyweight; Nippon Zenyaku Kogyo, Japan), midazolam (Sandoz; 2 mg·kg^−1^ bodyweight; Sandoz, Japan), and butorphanol tartrate (Vetorphale, 2.5 mg·kg^−1^ bodyweight; Meiji Seika Co., Japan). After clipping the leg of the rat, the skin surrounding the tibia was cut and the muscle was peeled. After exposing the tibia, the access hole (diameter: 1 mm) was prepared using VIVA ace (GC, Japan). The prepared titanium screw was implanted into the tibia of the rats with an average torque of 1.1 ± 0.2 cN·m. After implantation, the skin was rigidly sutured to prevent infection. The removal torque test of the implanted titanium screw was performed using Dejirache (No. GLK060, KTC, Japan) 28 days after surgery. All animal experimental protocols were reviewed and approved by the Institutional Animal Experiment Committee of Tohoku University (Approval number: 2018DnA-044) before beginning the animal experiments.

#### Histological observation

The tissue surrounding the implants was extracted and fixed using 4% glutaraldehyde. The samples were embedded into methylacrylate resin, cut into 5–7 μm sections after standardised dehydration and infiltration procedures, and stained with haematoxylin and eosin (H-E). The prepared sections were observed under a light microscope.

### Statistical analyses

All data are presented as means ± standard deviation (SD). Normal distribution of the data was verified using the Shapiro-Wilk test. Since data were normally distributed, statistical significance (p < 0.05) of each parameter for the surface characterisation of titanium specimens and the cell proliferation assay was assessed by either the Student’s t-test for pairwise comparisons or one-way analysis of variance (ANOVA), followed by the Tukey-Kramer honestly significant difference test for multiple comparisons using SPSS (IBM, USA).
